# The Association Between Trajectories of Self-reported Psychotic Experiences and Continuity of Mental Health Care in a Longitudinal Cohort of Adolescents and Young Adults

**DOI:** 10.1093/schbul/sbae136

**Published:** 2024-08-07

**Authors:** Suzanne E Gerritsen, Koen Bolhuis, Larissa S van Bodegom, Athanasios Maras, Mathilde M Overbeek, Therese A M J van Amelsvoort, Dieter Wolke, Giovanni de Girolamo, Tomislav Franić, Jason Madan, Fiona McNicholas, Moli Paul, Diane Purper-Ouakil, Paramala Santosh, Ulrike M E Schulze, Swaran P Singh, Cathy Street, Sabine Tremmery, Helena Tuomainen, Gwen C Dieleman, Esther Mesman

**Affiliations:** Department of Child and Adolescent Psychiatry and Psychology, Erasmus Medical Center, Rotterdam, the Netherlands; Department of Child and Adolescent Psychiatry and Psychology, Erasmus Medical Center, Rotterdam, the Netherlands; Department of Child and Adolescent Psychiatry and Psychology, Erasmus Medical Center, Rotterdam, the Netherlands; Yulius Mental Health Organization, Dordrecht, the Netherlands; ARQ National Psychotrauma Centre, Diemen, the Netherlands; ARQ National Psychotrauma Centre, Diemen, the Netherlands; Department of Clinical Child and Family Studies, Vrije Universiteit Amsterdam, Amsterdam, the Netherlands; Yulius Academy, Yulius Mental Health Organization, Dordrecht, the Netherlands; Department of Psychiatry and Neuropsychology, University of Maastricht, Maastricht, the Netherlands; Mondriaan Mental Health Care, Heerlen, the Netherlands; Department of Psychology, University of Warwick, Coventry, UK; Warwick Medical School, University of Warwick, Coventry, UK; IRCCS Istituto Centro San Giovanni di Dio Fatebenefratelli, Brescia, Italy; University Hospital Split, Split, Croatia; School of Medicine, University of Split, Split, Croatia; Warwick Clinical Trials Unit, Warwick Medical School, University of Warwick, Coventry, UK; School of Medicine & Medical Science, University College Dublin, Dublin, Republic of Ireland; Lucena CAMHS, SJOG, Dublin, Republic of Ireland; Warwick Medical School, University of Warwick, Coventry, UK; Coventry and Warwickshire Partnership NHS Trust, Coventry, UK; Centre Hospitalier Universitaire de Montpellier, Saint Eloi Hospital, Montpellier, France; INSERM, CESP U1018, PsyDev, University Paris Saclay, UVSQ, Versailles, France; Department of Child & Adolescent Psychiatry, Institute of Psychiatry, Psychology and Neuroscience, Kings College London, London, UK; Centre for Interventional Paediatric Psychopharmacology and Rare Diseases, South London and Maudsley NHS Foundation Trust, London, UK; HealthTracker Ltd, Kent, UK; Department of Child and Adolescent Psychiatry/Psychotherapy, University of Ulm, Ulm, Germany; Warwick Medical School, University of Warwick, Coventry, UK; Warwick Medical School, University of Warwick, Coventry, UK; Department of Neurosciences, KU Leuven, Leuven, Belgium; Warwick Medical School, University of Warwick, Coventry, UK; Department of Child and Adolescent Psychiatry and Psychology, Erasmus Medical Center, Rotterdam, the Netherlands; Department of Child and Adolescent Psychiatry and Psychology, Erasmus Medical Center, Rotterdam, the Netherlands

**Keywords:** adolescent, young adult, transition to adult care, psychotic disorders, mental health services, psychotic symptoms

## Abstract

**Background and Hypothesis:**

Young people (YP) with psychotic experiences (PE) have an increased risk of developing a psychiatric disorder. Therefore, knowledge on continuity of care from child and adolescent (CAMHS) to adult mental health services (AMHS) in relation to PE is important. Here, we investigated whether the self-reported trajectories of persistent PE were associated with likelihood of transition to AMHS and mental health outcomes.

**Study Design:**

In this prospective cohort study, interviews and questionnaires were used to assess PE, mental health, and service use in 763 child and adolescent mental health service users reaching their service’s upper age limit in 8 European countries. Trajectories of self-reported PE (3 items) from baseline to 24-month follow-up were determined using growth mixture modeling (GMM). Associations were assessed with auxiliary variables and using mixed models. ***Study results.*** At baseline, 56.7% of YP reported PE. GMM identified 5 trajectories over 24 months: medium increasing (5.2%), medium stable (11.7%), medium decreasing (6.5%), high decreasing (4.2%), and low stable (72.4%). PE trajectories were not associated with continuity of specialist care or transition to AMHS. Overall, YP with PE reported more mental health problems at baseline. Persistence of PE or an increase was associated with poorer outcomes at follow-up.

**Conclusions:**

PE are common among CAMHS users when reaching the upper age limit of CAMHS. Persistence or an increase of PE was associated with poorer mental health outcomes, poorer prognosis, and impaired functioning, but were less discriminative for continuity of care.

## Introduction

Psychiatric disorders emerging in childhood and adolescence are likely to persist into adulthood. Many children who receive mental health care are still expected to need care later as adults^1,2^ and have poorer functional outcomes compared with those with no history of psychiatric problems.^3^ Therefore, continuity of care for young people (YP) with mental health problems is of paramount importance. However, continuity of mental health care for adolescents and young adults with mental health problems is far from guaranteed: research shows that only 4% of child and adolescent mental health service (CAMHS) users experience an optimal transition from CAMHS to adult mental health services (AMHS)^4–7^ (although the term “transition” can be defined in different ways, in this manuscript the term “transition” is only used in the context of mental health care transition, not in the context of “transition” to a psychotic-spectrum disorder). Although YP with a formal diagnosis of a severe mental disorder, such as a psychotic-spectrum disorder, are likely to transition successfully to AMHS,^8–13^ this is less often the case for YP with a history of psychotic experiences (PE) or emotional disorders like depression or anxiety.^13–15^ Two studies^13,15^ showed that CAMHS users with PE were not more likely than CAMHS users without PE to receive continued treatment after reaching the upper age limit of their CAMHS. Another study^14^ showed that CAMHS users with PE were even less likely to be recommended to continue treatment by their CAMHS clinician. This is surprising, as PE are a potential risk marker for severe mental illness.^16,17^

Despite the potential severity, PE such as hallucinations and delusions during childhood and adolescence are common in the general population (17% of children aged 9–12 years and 7.5% of adolescents aged 13–18 years experience psychotic symptoms)^18^ and among (C)AMHS users (46%).^18–21^ Additionally, PE are often transient, self-resolving, and nonrecurring and do not signal subsequent psychopathology.^21,22^ Nevertheless, YP with PE have an increased risk of developing psychotic-spectrum disorders^23–27^ and nonpsychotic psychiatric disorders such as affective and anxiety disorders and suicidality,^28–30^ especially if PE are persistent during adolescence.^31^ Also, general population studies show an association between healthcare service use and PE during (pre)adolescence.^32,33^ Having received any outpatient or inpatient treatment at CAMHS in childhood or adolescence was associated with the risk of developing a psychotic disorder (2–13%) by the age of 28.^34^ As the period in which CAMHS users who need continued treatment have to transition to AMHS because of age restrictions, this is also the period in which most severe mental illnesses have their onset,^35^ monitoring of CAMHS users for the onset of PE and those with PE might therefore be important. Therefore, transition-related discontinuation of treatment^7^ or a delay in treatment^36^ for CAMHS users with PE may have negative long-term effects on mental health outcomes, associated with relapse and rehospitalization.^36^

To date, no studies have investigated the trajectories of PE (ie, persistence, intensity) and its associations with mental health care service use in the transition from CAMHS and AMHS. Within this context, it is also important to study risk factors for trajectories of PE and the development of psychotic-spectrum disorders or other severe mental illness. It is known that certain factors increase the risk of PE in the general population. These include the presence of (comorbid) psychopathology, substance use, traumatic life events including bullying, environmental stress, lower socioeconomic status (SES), and pre- and perinatal complications. Persistent PE are more often reported in women and those with substance use or markers of poorer mental health (eg, higher medication prescriptions). Findings on familial risk, urbanicity, and SES are less clear (for a comprehensive review see Staines et al^21^). A better insight into PE and these risk factors/indicators could guide CAMHS professionals in clinical decision-making regarding transition.^21,37^

In this study, we investigated self-reported trajectories of PE and potential indicators of severe mental health problems and psychotic-spectrum disorders in a clinical sample of YP reaching the upper age limit of CAMHS in Europe over a 24-month follow-up period. Moreover, we examined the association between these trajectories and continuity of specialist mental health care, specifically a transition to AMHS. Finally, we assessed differences in mental health outcomes and service use between these trajectories of PE as this could guide clinical decision making.

## Materials and Methods

### Study Design and Participants

This study was performed in the MILESTONE cohort,^38^ which included 763 service users from 39 CAMHS in 8 European countries (Belgium, Croatia, France, Germany, Italy, Ireland, the Netherlands, and the United Kingdom). These YP received care at CAMHS within 1 year before the upper age limit of their service (or 3 months after, if still in CAMHS). The upper age limit of the participating CAMHS was 18 years for two-thirds of services, or applied flexibly, varying between 16 and 19 years of age. YP did not have intellectual impairment and were able to complete questionnaires and interviews. A CAMHS care coordinator or clinician checked inclusion criteria and asked consent for the young person to be contacted by a MILESTONE research assistant who completed the consent procedure. The young person’s parent/carer (parents from hereon) and his/her clinician were also informed about the study and asked to consent to participate as informants. Supplementary figure 1 describes all exclusion criteria, the flow of participants from assessment for eligibility to recruitment, and participation at baseline and 9-, 15-, and 24-months follow-up. The current study describes a selection of 711 of 763 YP with at least one assessment of PE at any timepoint. The study protocol was approved (ISRCTN83240263; NCT03013595) by the UK National Research Ethics Service Committee West Midlands—South Birmingham (15/WM/0052) and ethics boards in participating countries.

### Procedure

Online questionnaires and interviews were used to assess sociodemographic and clinical characteristics from all informants. Assessments were face-to-face at the clinic at baseline and 24 months follow-up and via telephone at 9- and 15-months follow-up (or face-to-face upon request). Results of the assessment were not shared with the treating clinician. Detailed study procedures have been described more elaborately in Gerritsen et al.^38^

## Measures

### PE

PE were measured at all time points with the following 3 items from the Youth Self-Report^39^ (YSR; <18 years) and equivalents in the Adult Self-report^40^ (ASR; ≥18 years) as previously done by Bolhuis et al^41^: “I hear sounds or voices that according to other people are not there”; “I see things that other people think are not there”; and “I have thoughts that other people would think are strange.” YP indicated whether these statements were “not true” (0), “somewhat or sometimes true” (1), or “very true” (2) over the previous 6 months. We calculated the sum of the responses to these items to capture PE reported at each time point.

### Mental Health Service Use Indicators and Outcomes at Baseline

Mental health service use and care pathways. Mental health service use was assessed with the question: “Are you currently using a mental health service?” Response categories were: (1) CAMHS, (2) AMHS, or (3) no mental health services. “No mental health services” included response categories “no mental health services” and “other services,” that is, other than mental health services, such as community services. YP were categorized as having experienced “continued” or “discontinued” specialist care using self-reported mental health care use at all time points as follows (see Supplementary figure 2): YP who experienced continued specialist care had either transitioned to AMHS or remained in CAMHS. YP who experienced discontinued care either had their specialist care end or returned to care later.

Referral. At each assessment following an assessment that the young person was still in CAMHS, the CAMHS clinician was asked whether he/she had referred the young person to another service and if so, where to. Responses were categorized as “stay in CAMHS,” “referral to AMHS,” and “discharge.”

Visiting accident & emergency (A&E) department and visiting the GP. YP reported visits (yes/no) to the A&E department and/or the GP in the last 6 months on the Client Sociodemographic and Service Receipt Inventory EU version (CSSRI-EU).^42^

### Indicators and Outcomes Related to Mental Health and Psychotic-Spectrum Disorders

Clinical classifications based on ICD-10^43^ and DSM-IV^44^ and DSM-5^45^ were collected from CAMHS medical records. If no official classification was registered in the records, the preliminary classification was requested from the clinician. As the availability of clinical classifications was dependent on whether the young person received care, only clinical classifications registered at baseline were used.

Comorbidity was assessed as the number of clinical classifications registered.

Self- and parent-reported emotional/behavioral problems were assessed with the Y/ASR (self-reported) and parent-report versions of these questionnaires, the Child and Adult Behavior Checklist (C/ABCL)^39,40^ and include items relating to internalizing as well as externalizing problems. As these measures have different numbers of items, the mean of the item scores included in the “total problems scale” of the Y/ASR and C/ABCL was used. Mean item scores range from 0 to 2 and higher scores indicate more emotional and behavioral problems.

Clinician-rated severity of psychopathology was assessed using the Clinician Global Impression-Severity scale,^46^ which captures the severity of psychopathology over the last week relative to other YP with similar problems according to the CAMHS clinician. The scale ranges from “not at all ill” (score = 1) to “among the most extremely ill” (score = 7).

Self-reported self-harm and self-reported suicidal thoughts/behaviors were measured with two items from the Transition Readiness and Appropriateness Measure (TRAM) at baseline and the Transition Outcome Measure (TROM), the TRAM’s equivalent, at 24 months follow-up.^47^ Both the TRAM and TROM were specifically developed for the MILESTONE study. Self-harming behavior was assessed with the item “I have injured myself on purpose without intending to kill myself by cutting, scratching, burning, overdosing on pills, swallowing harmful objects/liquids or other methods” and suicidal thoughts and behaviors with “I have suicidal thoughts, wish I was dead, imagine how I would kill myself, and/or have attempted to end my own life.” Response categories were: (0) “rarely” (1), “sometimes” (2), “often” (3), “most of the time” (4), or “all of the time” (5).

Psychological quality of life was measured using the psychological quality of life domain of the World Health Organization Quality of Life Brief Inventory (WHOQOL-BREF),^48^ which assesses quality of life in the last 2 weeks. Mean domain scores were multiplied by 4 per scoring guidelines,^48^ giving a mean score ranging 4–20. Higher scores indicate a better quality of life.

Daily functional skills were measured using the specific levels of functioning (SLOF).^49^ Skills are rated across 6 domains: physical functioning, personal care, interpersonal relationships, social acceptability, activities, and work skills. Scores range from 1 to 5 and higher scores indicate better functioning.

Psychotropic medication use was assessed using the CSSRI-EU.^42^ Reported medication was recoded to generic drug names and categorized by the first author. For this study, the use of mood stabilizers and antipsychotics is reported.

Problems with peer relationships and problems with alcohol and substances were assessed with the Health of the Nation Outcome Scale for Children and Adolescents (HoNOSCA)^50^ domains “problems with peer relationships” and “problems with alcohol, substance/solvent misuse.” Based on semi-structured interviews with the young person, parents, and clinicians, a research assistant rated these problems over the last 2 weeks as not significant (0), transient or slight (1), mild but definite (2), moderate (3), or severe (4).

Parental psychopathology was assessed in the sociodemographic interview with parents. Research assistants asked the biological parent whether he/she or the other biological parent were “ever examined or treated for mental, developmental, language, speech or learning problems.” Responses were categorized as (1) psychopathology in one or both biological parent(s) or (2) no psychopathology. If the respondent was not a biological parent this information was coded as missing.

The number of life events experienced in the last 9 months was assessed using 13 items assessing different life events such as separation, accidents, and deaths.

Bullying victimology was assessed using a measure adapted from the Retrospective Bullying and Friendship Interview Schedule.^51^ YP indicated having been bullied at primary school, high school, at home, and at work or college (when applicable) and the frequency of the bullying: “never,” “once or twice,” “occasionally,” “about once a week,” “several times a week.” YP were scored as having been the victim of bullying if they indicated having been bullied occasionally or more often at one of the settings questioned.

### Sociodemographic Characteristics

Gender, the highest level of parent-reported parental education (dichotomized as primary/secondary/vocational and university), and self-reported educational/employment (dichotomized as being in school/working or not) were assessed at baseline.

## Missing Data

Table 1 describes the proportions of missing data for each measure. Multiple imputations in R (using mice^52^ and miceadds^53^) were applied to account for missing data under the assumption that data was “missing at random” (MAR). Previous analyses^38^ support this assumption, as missings were found to be dependent on observed values. All variables included in the analyses described in this manuscript were imputed, except for self-reported PE as Mplus deals with missing data in the outcome measure in growth mixture modeling (GMM) using maximum likelihood under MAR. The variable “site” (indicating the CAMHS in which the young person was recruited) was used as a cluster variable. The data was imputed with the default method in mice, after which density plots and trace lines were used to inspect the imputations and convergence respectively. Following these inspections, the method of imputation was changed per variable, to see whether other methods improved the imputation.

**Table 1.** Sample Descriptives and Proportions of Missing Data (*n* = 711)

	T1		T4	
	*M* (SD) /Median [IQR] or *n* (%)	*N* (%) missing data	*M* (SD) /Median [IQR] or *n* (%)	*N* % missing data
Sociodemographic characteristics				
Age	17.5 (0.59)	7 (1.0%)	NA	NA
Gender (female)	431 (60.6%)	0 (0.0%)	NA	NA
Country				
Belgium	64 (9.0%)	0 (0.0%)	NA	NA
Croatia	52 (7.3%)	0 (0.0%)	NA	NA
France	77 (10.8%)	0 (0.0%)	NA	NA
Germany	55 (7.7%)	0 (0.0%)	NA	NA
Ireland	37 (5.2%)	0 (0.0%)	NA	NA
Italy	163 (22.9%)	0 (0.0%)	NA	NA
Netherlands	109 (15.3%)	0 (0.0%)	NA	NA
United Kingdom	154 (21.7%)	0 (0.0%)	NA	NA
Parental educational level (high)	194 (27.3%)	159 (22.4%)	NA	NA
Being in school or working	625 (87.9%)	33 (4.6%)	417 (58.6%)	224 (31.5%)
PE				
Self-reported PE	1.00 [0.00, 2.00]	28 (3.9%)	0.00 [0.00, 1.00]	233 (32.8%)
Mental health service use indicators and outcomes				
Self-reported mental health service use				213 (30.0%)
CAMHS	NA	NA	62 (8.7%)	
AMHS	NA	NA	71 (10.0%)	
Other	NA	NA	54 (7.6%)	
No MHS	NA	NA	311 (43.7%)	
Clinician-reported referral between baseline and 9months f-u		142 (20.0%)		
Stay in CAMHS	213 (30.0%)		NA	NA
Referral to AMHS	107 (15.0%)		NA	NA
Discharge	249 (35.0%)		NA	NA
Self-reported visits to A&E department (CSSRI-EU)	84 (11.8%)	49 (6.9%)	43 (6.0%)	225 (31.6%)
Self-reported visits to GP (CSSRI-EU)	275 (38.7%)	49 (6.9%)	220 (30.9%)	225 (31.6%)
				
Indicators and outcomes related to mental health and psychotic-spectrum disorders				
Clinical classification: Bipolar disorder (yes)	15 (2.1%)	0%	NA	NA
Clinical classification: Schizophrenia spectrum disorder (yes)	32 (4.5%)	0%	NA	NA
Comorbidity (number of clinical classifications; yes)	1.00 [1.00, 2.00]	0 (0%)	NA	NA
Self-reported emotional/behavioral problems (Y/ASR; range 0–2)	0.56 (0.28)	28 (3.9%)	0.47 (0.30)	233 (32.8%)
Parent-reported emotional/behavioral problems (C/ABCL; range 0–2)	0.39 (0.24)	150 (21.1%)	0.42 (0.28)	335 (47.1%)
Clinician-rated severity of psychopathology (CGI-S; range 1–7)	3.42 (1.39)	102 (14.3%)	NA	NA
Self-reported suicidal thoughts/behaviors (TRAM/TROM; range 0–5)	0.00 [0.00, 2.00]	18 (2.5%)	0.00 [0.00, 1.00]	239 (33.6%)
Self-reported self-harm (TRAM/TROM; range 0–5)	0.00 [0.00, 1.00]	18 (2.5%)	0.00 [0.00, 0.00]	239 (33.6%)
Self-reported psychological quality of life (WHOQOL-BREF; range 4–20)	12.04 (3.54)	26 (3.7%)	13.06 (3.41)	221 (31.1%)
Parent-reported daily functional skills (SLOF; range 1–5)	4.47 [4.12, 4.72]	145 (20.4%)	NA	NA
Self-reported use of antidepressants (yes; CSSRI-EU)	216 (30.4%)	49 (6.9%)	110 (15.5%)	225 (31.6%)
Self-reported use of mood stabilizers (yes; CSSRI-EU)	18 (2.5%)	49 (6.9%)	25 (3.5%)	225 (31.6%)
Self-reported use of antipsychotics (yes; CSSRI-EU)	96 (13.5%)	49 (6.9%)	59 (8.3%)	225 (31.6%)
Problems with peer relationships (HoNOSCA; range 0–4)	0.00 [0.00, 1.00]	19 (2.7%)	NA	NA
Problems with alcohol and substances (HoNOSCA; range 0–4)	0.00 [0.00, 1.00]	20 (2.8%)	NA	NA
Parent-reported parental psychopathology (yes)	191 (26.9%)	182 (25.6%)	NA	NA
Number of life events (range 0–13)	2.00 [1.00, 3.00]	31 (4.4%)	NA	NA
Bullying victimology (yes)	426 (59.9%)	30 (4.2%)	NA	NA

*Note*: NA = not applicable or not assessed.

## Statistical Analyses

Trajectories of self-reported PE from baseline to 24 months follow-up were determined using Growth Mixture Modelling in Mplus. The best-fitting model was determined based on fit indices Aikaike Information Criterion (AIC), Bayesian Information Criterion (BIC), entropy, Bootstrapped Likelihood Ratio Test (BLRT), Vuong–Lo–Mendell–Rubin Likelihood Ratio (VLMR) test, Lo–Mendell–Rubin Likelihood Ratio (LMR) test, and the number of YP in each class and posterior probabilities, according to Jung and Wickrama.^54^

After determining the best-fitting model, the associations between care pathways and developmental trajectories of PE were assessed by including care pathways as auxiliary variables in the growth mixture model. We corrected for parental educational level and gender due to their potential confounding effect in the relationship between care pathways and PE. As including country (with 8 categories) in the models, as a covariate resulted in convergence problems, country was not included in the final models. Odds ratios and 95% confidence intervals are presented. We assessed the association between trajectories of PE and care pathways dichotomized in 2 ways: continued versus discontinued specialist care and continued care in CAMHS versus continued care in AMHS. As a sensitivity analysis, we also assessed associations between trajectories of PE and the 4 pathways: “transition,” “remain in CAMHS,” “return to care,” and “end of care” (= reference category). Second, the following mental health indicators were assessed (as auxiliary variables): referral, clinical classifications, comorbidity, clinician-rated severity of psychopathology (CGI-S), self-reported suicidal thoughts/behaviors, and self-harm (TRAM/TROM), parent-reported daily functional skills (SLOF), problems with peer relationships and problems with alcohol and substances (HoNOSCA), parental psychopathology, the number of life events and bullying victimology.

Finally, associations between trajectories of PE and the following outcomes were assessed: self-and parent-reported emotional/behavioral problems (Y/ASR and C/ABCL), self-reported suicidal thoughts/behaviors and self-harm (TRAM/TROM), self-reported psychological quality of life (WHOQOL-BREF), self-reported visits to A&E department and the GP (CSSRI-EU), self-reported use of antipsychotics and mood stabilizers (CSSRI-EU), and being in school or working. These associations were assessed using linear (for continuous outcomes) and logistic (for dichotomous outcomes) mixed models using lme4^55^ in R version 3.6.3, with a variable indicating the CAMHS of recruitment as a random effect. Models were fitted separately for each outcome as a dependent variable, with a variable indicating the most likely PE trajectory for each individual included as an independent variable. Baseline levels of the outcome variables (ie, baseline Y/ASR scores when assessing Y/ASR scores at 24 months follow-up), parental educational level, gender, and country were also included as fixed effects. If the assumption of heteroscedasticity was violated, the outcome variables were transformed. Estimated marginal means/proportions and corresponding 95% confidence intervals were presented for the full models.

## Results

### Descriptives

Table 1 provides descriptive characteristics of the 711 YP included in this study at baseline and 24 months follow-up using observed data. On average, YP were 17.5 years old (SD = 0.59). At baseline, 403 subjects (56.7%) had a PE score of 1 or higher, whereas 280 YP (39.4%) reported no PE (information was missing for *n* = 28, 3.9%). After multiple imputations, care pathways were determined. In total, 46.5% of YP had experienced continuity of specialist care (25.7% staying in CAMHS and 20.8% transitioning to AMHS) and 53.5% of YP had experienced discontinuity of care (40.6% of YP experiencing end of care and 12.9% returning to care).

### Developmental Trajectories of PE

**Fig. 1.**
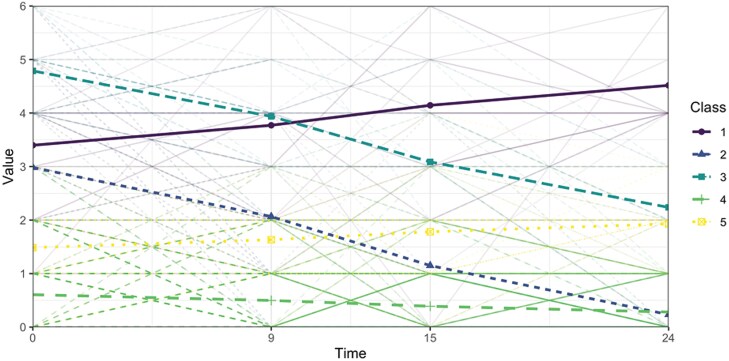
Five latent classes representing developmental trajectories of self-reported PE. *Note*: Class 1 (medium increasing): *n* = 37 (5.2%); class 2 (medium decreasing): *n *= 46 (6.5%); class 3 (high decreasing): *n *= 30 (4.2%); class 4 (low stable): *n *= 515 (72.4%); class 5 (medium stable): *n *= 83 (11.7%).

Seven Growth Mixture Models of trajectories of self-reported PE were fitted with 1 up to 7 classes. Supplementary table 1 presents model fit indices for these models. The 5-class model was determined to have the best fit. Several indices showed that models with more classes fit the data better: the AIC and BIC decreased with an increasing number of latent classes and the BLRT, which compares the fit of the model with k classes versus a model with k-1 classes, was statistically significant up to the 7-class model. However, the VLMR test indicated a 7-class model was not superior to a 6-class model, and the number of YP per class decreased considerably from a 6-class model onwards. Latent classes smaller than *n* = 30 were considered too small for conducting subsequent analyses, that is, assessing relationships with predictors and outcomes. The entropy of 0.83 indicated a good classification accuracy of the 5-class model. A 5-class model with a quadratic or cubic slope showed comparable fit, so the most basic (linear) 5-class model was chosen. Figure 1 graphically depicts the developmental trajectories of PE according to the best-fitting model. The five classes were defined as follows: class 1 (medium increasing): *n* = 37 (5.2%); class 2 (medium decreasing): *n* = 46 (6.5%); class 3 (high decreasing): *n* = 30 (4.2%); class 4 (low stable): *n* = 515 (72.4%); and class 5 (medium stable): *n* = 83 (11.7%). Taken together, most YP reporting PE show decreasing or low stable trajectories (class 2, 3, 4 = 83.1%).

### Associations Between Developmental Trajectories of PE and Care Pathways

Associations between care pathways as auxiliary variables and developmental trajectories of PE were assessed in the growth mixture model. Table 2A shows the proportions of YP in the different trajectories of PE and their care pathways. End of care was 40.6% on average and least likely in the “high decreasing” group (21.9%). On average 12.9% returned to care in the follow-up period. Table 2B presents odds ratios and corresponding 95% confidence intervals describing the association between trajectories of PE and care pathways. The trajectories of self-reported PE were not associated with continuity of specialist care (vs discontinued specialist care). Continuity of specialist care was most likely in the “high decreasing” group (continuity of specialist care OR 2.83; CI 0.94–8.53). Of note, power was limited due to the small group size and, hence, *P*-values were not calculated. Looking at transition to AMHS (vs staying in CAMHS), the confidence interval for the odds of YP in a medium-decreasing trajectory to transition to AMHS was very large, suggesting separation due to small proportions, which affects the reliability of this result. Analyses were repeated with the four care pathways (see supplementary table 2). Again, no relationships between trajectories of PE and these four care pathways were found. This analysis also resulted in large confidence intervals, specifically for the odds of YP in the medium-increasing trajectory to return to care.

**Table 2.** Associations Between Developmental Trajectories of PE and Care Pathways

	Developmental Trajectories of Self-reported PE									
	Low stable (c4)	Medium stable (c5)		Medium increasing (c1)		Medium decreasing (c2)		High decreasing (c3)		Total
	(*N* = 515, 72.4%)	(*N* = 83; 11.7%)		(*N* = 37; 5.2%)		(*N* = 46; 6.5%)		(*N* = 30; 4.2%)		(*N* = 711, 100%)
A. Proportions of young people with trajectories of PE following different care pathways										
Care pathways	Prop. (%)	Prop. (%)		Prop. (%)		Prop. (%)		Prop. (%)		Prop. (%)
End of care	42.4	32.6		37.5		49.0		21.9		40.6
Return to care	12.8	12.3		11.3		17.5		11.7		12.9
Transition to AMHS	18.9	25.5		35.9		14.9		29.5		20.8
Remain in CAMHS	25.8	29.6		15.3		18.6		36.9		25.7
Total	100.0	100.0		100.0		100.0		100.0		100.0
B. Odds ratios describing the association between developmental trajectories of PE and care pathways										
		OR	95% CI	OR	95% CI	OR	95% CI	OR	95% CI	
Care pathways: continued care vs discontinued care (ref.)	-	1.62	0.90–2.91	1.18	0.46–3.03	0.44	0.13–1.48	2.83	0.94–8.53	
Care pathways: AMHS vs CAMHS (ref.)	-	1.51	0.51–2.62	8.89	0.00–34,291.27	0.00	0.00–*****	1.04	0.34–3.21	

*Note:* A. Column proportions are presented. For the purpose of this cross-tabulation, YP were assigned to the likely developmental trajectory using posterior probabilities. Total *N* for developmental trajectories is 711, 93.2% of the total cohort, as the developmental trajectories were only estimated for those with information about PE from at least one assessment. B. Growth mixture model with several auxiliary models (three-step approach). Models are corrected for gender and parental highest education level.

As power may be an issue, we also performed subsequent logistic regression analyses in which groups of participants were distinguished based on previously used methods (eg, ^22,56^). This approach resulted in the following 4 groups: none/low (T1–T4 ≤ 1; proportion 44%), incident/increasing (T1 ≤ 1/T4 ≥ 2; proportion 27%), remittent (T1 ≥ 2/T4 ≤ 1; proportion 21%), and persistent (T1–T4 ≥ 2; proportion 8%) PE. In subsequent logistic regression analyses, we found that the persistent PE group was more likely to continue specialist care (OR = 2.97, SE = 0.115, *P* = .008). However, we did not find that persistent PE were associated with a greater likelihood for transition to AMHS versus staying in CAMHS specifically (OR = 1.40, SE = 0.439, *P* = .702). Importantly, these groupings were not directly comparable with GMM-based classes, but these analyses suggest that continuity of specialist care and PE trajectories might be associated if PE are persistent.

### Associations Between Indicators of Mental Health, Mental Health Service Use, and Developmental Trajectories of PE

Odds ratios and confidence intervals describing associations between developmental trajectories of PE and indicators of mental health service use and mental health (as auxiliary variables in the growth mixture model) are presented in table 3. Transition to AMHS was not associated with PE at baseline, results in table 3 show that CAMHS clinicians were more likely to indicate that YP in the medium stable trajectory needed to be referred to AMHS, compared to those in the low stable trajectory. Notwithstanding, although not statistically significant, the other trajectories report increased referrals as compared to the low stable trajectory and confidence intervals between these trajectories overlapped with the medium stable trajectory. Here, the PE classes which resulted in small group size for some categories possibly caused related power issues. We also found that, overall, YP with PE (medium stable/increasing/ decreasing and high decreasing) reported more mental health problems than YP in the low stable trajectory. All four groups of YP with PE reported more suicidal thoughts/behaviors (TRAM/TROM) than YP in the low stable trajectory. YP with medium increasing/decreasing and high decreasing PE, thus “medium” of “high” scores at baseline, reported more self-harm (TRAM/TROM), and more life events and parents of these YP reported less daily functional skills (SLOF) than those with low stable PE. YP with medium stable/increasing/decreasing PE reported more problems with peer relationships (HoNOSCA) and YP with medium stable/increasing PE reported more problems with alcohol and substances (HoNOSCA). For more differences between specific latent classes, see table 3.

**Table 3.** Association Between Developmental Trajectories of PE, Mental Health Service Use Indicators, and Indicators of Mental Health and Severe Mental Illness at Baseline

	Developmental Trajectories of Self-reported PE								
	Low stable (c4)	Medium stable (c5)		Medium increasing (c1)		Medium decreasing (c2)		High decreasing (c3)	
	(*N* = 515, 72.4%)	(*N* = 83; 11.7%)		(*N* = 37; 5.2%)		(*N* = 46; 6.5%)		(*N* = 30; 4.2%)	
		OR	95% CI	OR	95% CI	OR	95% CI	OR	95% CI
Auxiliary variables									
Mental health service use indicators									
Referral between baseline and 9 months f-u									
Stay in CAMHS (ref. cat. = Discharge)		1.38	0.69–2.77	1.26	0.41–3.84	0.65	0.20–2.13	1.62	0.55–4.82
Referral to AMHS (ref. cat. = Discharge)		**2.37**	**1.08–5.18**	2.11	0.64–7.00	0.86	0.20–3.77	1.91	0.51–7.24
Indicators of mental health and psychotic-spectrum disorders									
Clinical classification: Bipolar disorder		2.09	0.28–15.42	1.45	0.05–47.25	**7.83**	**1.24–49.35**	5.54	0.88–34.95
Clinical classification: Schizophrenia spectrum disorder		1.32	0.32–5.42	0.48	0.02–11.27	0.89	0.08–9.94	1.58	0.32–7.87
Comorbidity (number of clinical classifications)		1.33	0.92–1.92	1.02	0.63–1.66	1.23	0.67–2.29	**1.95**	**1.30–2.93**
Clinician-rated severity of psychopathology (CGI-S)		1.22	0.98–1.51	**1.54**	**1.01–2.35**	0.98	0.71–1.36	1.32	0.90–1.93
Self-reported suicidal thoughts/behaviors (TRAM/TROM)		**1.63**	**1.23–2.16**	**2.46**	**1.78–3.39**	**2.42**	**1.76–3.34**	**2.58**	**1.86–3.58**
Self-reported self-harm (TRAM/TROM)		1.33	0.97–1.82	**2.00**	**1.46–2.75**	**1.86**	**1.22–2.83**	**2.29**	**1.72–3.06**
Parent-reported daily functional skills (SLOF)		0.49	0.23–1.07	**0.26**	**0.10–0.68**	**0.32**	**0.14–0.76**	**0.33**	**0.16–0.69**
Problems with peer relationships (HoNOSCA)		**1.45**	**1.14–1.84**	**1.71**	**1.20–2.42**	**1.45**	**1.06–1.98**	1.24	0.84–1.83
Problems with alcohol and substances (HoNOSCA)		**1.57**	**1.07–2.29**	**2.09**	**1.28–3.43**	1.38	0.78–2.42	1.59	0.95–2.67
Parental psychopathology		1.18	0.61–2.30	1.53	0.54–4.36	2.50	0.84–7.41	1.83	0.72–4.65
Number of life events		1.14	0.97–1.33	**1.31**	**1.08–1.61**	**1.26**	**1.06–1.51**	**1.41**	**1.15–1.73**
Bullying victimology		1.81	0.97–3.40	2.59	0.82–8.17	7.19	0.90–57.60	**3.84**	**1.19–12.42**

*Note:* Growth mixture model with several auxiliary models (three-step approach). Models are corrected for gender and parental highest education level.

### Associations Between Trajectories of PE and Outcomes

Table 4 shows the associations between trajectories of PE and mental health outcomes. At 24 months follow-up, YP with medium stable PE reported more emotional/behavioral problems (Y/ASR), more suicidal thoughts/behaviors (TRAM/TROM), and less psychological quality of life (WHOQOL-BREF) than YP with medium decreasing, high decreasing, and low stable PE. YP with medium stable PE also reported more self-harm (TRAM/TROM) than YP with low stable PE. Similarly, YP with medium increasing PE reported more emotional/behavioral problems, more suicidal thoughts/behaviors and self-harm, and less psychological quality of life than YP with medium decreasing and low stable PE. YP with medium increasing PE also reported less psychological quality of life than YP with high decreasing PE. We found no association between PE trajectories and self-reported GP visits or visits to accident and emergency departments. There was also no difference between trajectories in school or work attendance. The results of the model assessing the association between PE trajectories and self-reported use of mood stabilizers (CSSRI-EU) at 24 months follow-up were considered unreliable due to large standard errors, as groups were too small, and are therefore not presented.

**Table 4.** Association Between Developmental Trajectories of PE and Mental Health Outcomes

		Developmental Trajectories of Self-reported Psychotic-like Experiences				
		Low stable (c4)	Medium stable (c5)	Medium increasing (c1)	Medium decreasing (c2)	High decreasing (c3)
		(*N* = 515, 72.4%)	(*N* = 83; 11.7%)	(*N* = 37; 5.2%)	(*N* = 46; 6.5%)	(*N* = 30; 4.2%)
	*P*-value for test of different means	Mean (95%CI)	Mean (95%CI)	Mean (95%CI)	Mean (95%CI)	Mean (95%CI)
Outcome variables						
Self-reported emotional/behavioral problems (Y/ASR; transformed to y3*)	**<.001**	**−1.45**1^,^4 **(−1.54 to −1.36)**	**−0.89** ^1,2,3^ **(−1.07 to −0.72)**	**−1.03**4^,^5 **(−1.30 to −0.75)**	**−1.72**2,5 **(−1.96 to −1.48)**	**−1.50**3 **(−1.81 to −1.20)**
Parent-reported emotional/behavioral problems (C/ABCL; transformed to y3*)	.246	**−**1.62 (**−**1.77 to −1.47)	**−**1.22 (**−**1.47 to −0.97)	**−**1.46 (**−**1.83 to **−**1.08)	**−**1.65 (**−**1.98 to **−**1.33)	**−**1.64 (**−**2.03 to **−**1.24)
Self-reported suicidal thoughts/behaviors (TRAM/TROM)	**<.001**	**0.16**1^,^4 **(0.13 to 0.21)**	**0.48** ^1,2,3^ **(0.36 to 0.61)**	**0.42**4^,^5 **(0.25 to 0.62)**	**0.14**2^,^5 **(0.07 to 0.27)**	**0.17**3 **(0.08 to 0.33)**
Self-reported self-harm (TRAM/TROM)	**<.001**	**0.10** ^1,2^ **(0.07 to 0.13)**	**0.25** ^¹^ **(0.17 to 0.37)**	**0.42**2,3 **(0.25 to 0.61)**	**0.14**3 **(0.07 to 0.25)**	**0.19 (0.09 to 0.36)**
Self-reported psychological quality of life (WHOQOL-BREF)	**<.001**	**13.40**1^,^4 **(13.05 to 13.75)**	**11.76** ^1,2,3^ **(11.09 to 12.43)**	**11.44** ^4,5,6^ **(10.42 to 12.46)**	**13.72**2^,^5 **(12.82 to 14.61)**	**13.74**3^,^6 **(12.63 to 14.85)**
Self-reported visits to A&E department (CSSRI-EU)	.890	0.09 (0.06 to 0.14)	0.13 (0.06 to 0.25)	0.14 (0.05 to 0.32)	0.09 (0.04 to 0.21)	0.11 (0.04 to 0.25)
Self-reported visits to GP (CSSRI-EU)	.790	0.41 (0.35 to 0.47)	0.49 (0.37 to 0.62)	0.42 (0.26 to 0.61)	0.39 (0.25 to 0.56)	0.51 (0.31 to 0.71)
Self-reported use of antipsychotics (yes; CSSRI-EU)	.073	0.13 (0.10 to 0.18)	0.25 (0.15 to 0.39)	0.38 (0.19 to 0.60)	0.31 (0.16 to 0.52)	0.17 (0.07 to 0.38)
Being in school or working	.274	0.90 (0.84 to 0.93)	0.87 (0.76 to 0.94)	0.76 (0.57 to 0.88)	0.85 (0.70 to 0.93)	0.81 (0.62 to 0.92)

*Note*: Models include the following covariates: baseline levels of these outcomes, country, parental educational level, and gender. Results are averaged over the levels of gender, country, and parental educational level. SE, standard error.

^1,2,3,4,5,6^Groups with the same superscripts differ statistically from one another (*P* < .05).

*If model assumptions were violated (see the statistical analysis section), the following transformations were applied: y1 = y/ymax (this was the first transformation we applied to y, with y starting at 0 and ymax as the maximum value y can take), y2 = (y1 × [*n* − 1] + 0·5)/*n*, and y3 = log[y2/(1 − y2)]), with y being the outcome. After each transformation the model assumptions were reassessed.

## Discussion

The aim of this study was to describe self-reported trajectories of PE, specialist care trajectories, and their association with potential indicators and outcomes of severe mental health problems in a clinical sample of CAMHS at the transitional ages toward adulthood. By doing so, we aimed to integrate the challenges of transition to specialist mental health care and mental health care problems in a period in which severe mental disorders may continue or further progress in which PE may be an important risk marker.

In line with previous research,^18,19^ we found that approximately half of all YP in CAMHS reported PE. GMM revealed five different trajectories of self-reported PE during this transitional period. Two trajectories can be considered as characterizing persistence of PE: the “medium stable” and “medium increasing” trajectories, whereas PE in the “high decreasing” group can be considered “transient.” These trajectories are similar (although not the same) to several other studies employing growth mixture models^57,58^ and overlap with the patterns of persistence as described in the meta-analysis by Staines and colleagues.^22^ It should, however, be noted that the majority of earlier studies have been performed in general population samples, which are not directly generalizable to our clinical sample, and this might explain the differences observed in a number of trajectories. Overall, one in six (16.9%) YP in our sample experienced persistent PE during a 24-month follow-up period in late adolescence, which is lower than in some studies,^22^ but in line with others.^59,60^ Definition of PE, methodological differences and age also may be at play here.^22^

Next, our study aimed to explore the association between PE trajectories and mental health characteristics to guide CAMHS professionals in the process of clinical decision-making regarding transition. In line with prior work,^21^ YP with persistent and increasing patterns of PE reported more mental health problems (eg, self-harm, suicidality, daily life functioning) at baseline and follow-up than YP in the low stable trajectory. Our findings are in line with Steenkamp et al^37^ who also established poor discrimination between persistent and transient PE. Notwithstanding, PE are common in the general pediatric population—and even more common in clinical populations, but—reassuringly—they are mostly transient in nature. In clinical populations, PE might warrant clinical follow-up when presenting in older adolescence or with one or more psychiatric diagnosis.^30^ Furthermore, PE that are persistent over time, distressing and impairing functioning might also require more clinical attention.^37,56^ Therefore, if present, PE deserve clinical exploration. In our sample, however, almost half of CAMHS users with persistent PE did not receive continued specialist mental health care, limiting the opportunity for follow-up.

Regarding care trajectories, our primary analyses showed that trajectories of PE were not related to continuity of specialist care in the period after reaching the CAMHS upper age limit and this is in line with previous findings.^13,15^ Additional analyses suggest that persistent PE are possibly associated with continuity of specialist care. Previous analyses on MILESTONE data indicated that CAMHS users with PE were less likely to be recommended to continue specialist treatment.^14^ The discrepancy between these studies may be explained by the fact that the current study assessed PE over a 24-month period rather than at baseline alone. We did find, however, that CAMHS clinicians were more likely to refer YP with medium stable PE to AMHS. It may be that the number of YP following other trajectories was too small to find statistically significant results. Alternatively, as noted above, CAMHS clinicians may not always be aware of their patients’ PE.^14^ The indication for referral may have been based on other mental health indicators, such as suicidality, for example. Another explanation may be that mental health problem levels in general, or the presence of severe and acute mental health problems such as suicidality, are stronger predictors of transition.^13,14^ The lack of a clear association between PE trajectories and care trajectories can be of concern, considering the association with mental health problems after 24 months. Notably, trajectories of PE were not associated with self-reported GP or A&E visits and participation in school or work at follow-up. Nonetheless, the presence of PE specifically should therefore be considered a “warning signal” and justifies follow-up by the clinician. Findings underscore the importance of awareness and exploration of options for continued monitoring in “low-threshold” services for those with persistent PE.^61^

The current study should be interpreted in the context of the following in strengths and limitations. Strengths of the current study include the use of GMM to empirically establish trajectories of PE within a clinical sample of 711 adolescents in the period they reach adulthood. Further, with the broad range of variables assessed within the MILESTONE study, we were able to describe characteristics of YP with persistent PE while still in CAMHS, their care pathways and their mental health outcomes after a 24-month follow-up period. There are also several limitations to the current study. First, a selection bias possibly affects the representativeness of the MILESTONE cohort. In earlier work, we illustrated that the MILESTONE cohort is comparable to other community clinical samples.^38^ However, of all YP that were not recruited, approximately 20% were not introduced to the study because the clinician or care coordinator deemed them to be too unwell to participate. However, these excluded participants were more likely to report PE, and that their exclusion are baseline may have biased the estimation of PE trajectories.^21^ Second, the number of YP in several PE trajectories was small resulting in lower statistical power. Third, by using the most likely PE trajectory for each individual as a categorical variable to assess differences on mental health outcomes and service use after 24 months follow-up, the uncertainty of the estimation of the most likely class was not accounted for. This may have increased the likelihood of finding statistical differences between classes. Fourth, the absence of clinical classifications for YP who were not in care at follow-up assessments meant we could not assess if and when YP’s PE developed into clinical psychotic disorders. This remains a recommendation for future research. Brief self-report measures of PE based upon, for example, the Y/ASR, despite being commonly used, brings up several challenges.^21^ In general, self-reported measures of PE are known to be nonspecific cross-sectionally, which could also explain our lack of association with clinical schizophrenia spectrum disorders, but can rather be considered a risk marker for future psychopathology, distress, and loss of functioning. For the Y/ASR specifically, the items used to assess PE are also included in the Y/ASR total scale assessing self-reported emotional/behavioral problems. Although this only regard 3 out of 104 (YSR) and 120 (ASR) items, the selection of these items may have resulted in a marginal overestimation of the association between PE and self-reported emotional/behavioral problems. Among the 3 items from the Y/ASR the item “I have thoughts that other people would think are strange” was included to capture the full range of PE including delusions. We recommend replication of this study using questionnaires or clinical interviews specifically developed to assess PE, as well as the use of multiple informants.^18,21^ Also, other mental health indicators not considered in the present study are potentially important for the identification of trajectories. Finally, we did not specifically investigate use of other (nonspecialist) services, such as community, social, and occupational services.

In conclusion, this study confirms earlier studies that PE are common, often transient, but can be persistent among CAMHS users during the transitional stage. Increasing or persisting PE trajectories were not clearly related to continuity of specialist care at 24 months follow-up, but persistence or an increase of PE was associated with poorer mental health outcome, poorer prognosis, impaired functioning, and increased mental health service use. Follow-up of PE and mental health indicators remains important when adolescents become adults and leave CAMHS.

## Supplementary Material

Supplementary material is available at https://academic.oup.com/schizophreniabulletin.

sbae136_suppl_Supplementary_Material

## Data Availability

The participant consent forms restrict sharing of data outside the MILESTONE consortium. All analyses were conducted in R. Code can be made available upon request to the corresponding author.

## Data Sharing Statement

The participant consent forms restrict sharing of data outside the MILESTONE consortium. All analyses were conducted in R. Code can be made available upon request to the corresponding author. The study protocol and statistical analysis plan is available at https://milestone-transitionstudy.eu/.
