# Supplementary material for: The Association Between Trajectories of Self-reported Psychotic Experiences and Continuity of Mental Health Care in a Longitudinal Cohort of Adolescents and Young Adults
Source: Schizophr Bull. 2024 Aug 7;51(5):1282–95. doi: 10.1093/schbul/sbae136 (PMC12414552; doi:10.1093/schbul/sbae136)
Supplement: sbae136_suppl_Supplementary_Material [file sbae136_suppl_supplementary_material.docx]

Supplementary Figure 1 – CONSORT Flow Diagram of participants

Note. YP = Young person, PC = parent/carer, CL = clinician.

Supplementary Figure 2 – Care pathways


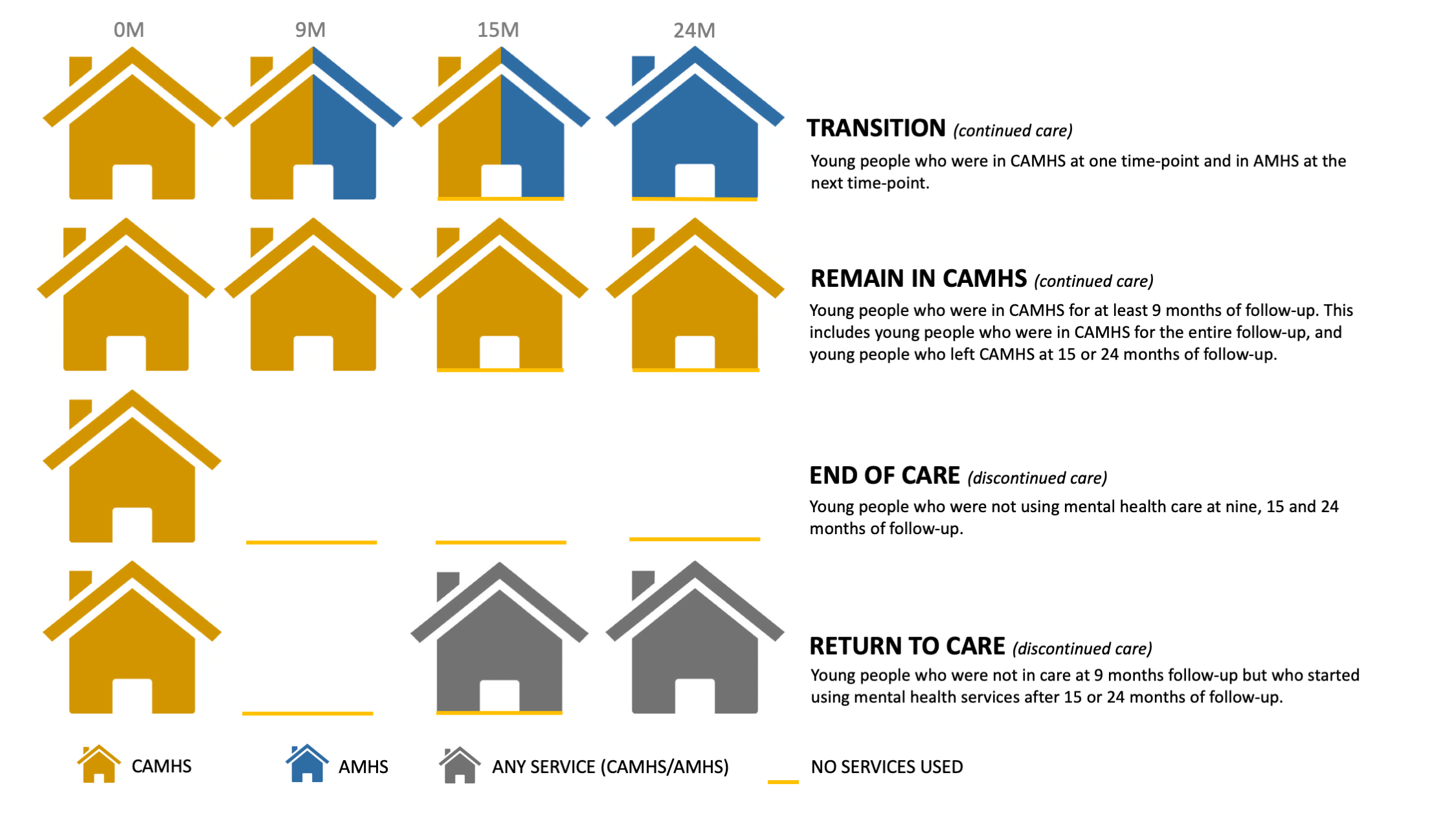


Note. Adaption of figure published previously in Appendix of paper published in Lancet Psychiatry (Gerritsen SE, van Bodegom LS, Overbeek MM, et al. Leaving child and adolescent mental health services in the MILESTONE cohort: a longitudinal cohort study on young people's mental health indicators, care pathways, and outcomes in Europe. *The Lancet Psychiatry*. 2022/12/01/ 2022;9(12):944-956. doi:<https://doi.org/10.1016/S2215-0366(22)00310-8>).

Supplementary Table 1 - Model fit for latent classes of PE (n = 711)

| Model | Number of classes | AIC | BIC | Entropy | p-value BLRT | p-value VLMR | p-value LMR | N in smallest class | N in largest class | Posterior prob. (lowest) | Posterior prob. (highest) |
| --- | --- | --- | --- | --- | --- | --- | --- | --- | --- | --- | --- |
| 1 | 1 | 6745.262 | 6777.229 | - | - | - | - | 711 | 711 | 1 | 1 |
| 2 | 2 | 6523.112 | 6568.779 | 0.920 | < 0.001 | 0.0042 | 0.0052 | 71 | 640 | 0.984 | 0.933 |
| 3 | 3 | 6427.985 | 6487.352 | 0.907 | < 0.001 | 0.0071 | 0.0086 | 42 | 622 | 0.794 | 0.973 |
| 4 | 4 | 6380.565 | 6453.631 | 0.828 | < 0.001 | 0.7361 | 0.7476 | 33 | 536 | 0.797 | 0.935 |
| **5** | **5** | **6325.914** | **6412.681** | **0.829** | **< 0.001** | **0.0301** | **0.0329** | **30** | **515** | **0.690** | **0.919** |
| 6 | 6 | 6279.592 | 6380.059 | 0.834 | < 0.001 | 0.0177 | 0.0206 | 5 | 483 | 0.699 | 0.998 |
| 7 | 7 | 5097.667 | 5211.834 | 0.832 | < 0.001 | 0.4055 | 0.4222 | 12 | 409 | 0.735 | 1.000 |

Note. AIC = Aikaike Information Criterion, BIC = Bayesian Information Criterion, BLRT = bootstrapped likelihood ratio test, VLMR = Vuong–Lo–Mendell–Rubin Likelihood Ratio Test, LMR = Lo–Mendell–Rubin Likelihood Ratio Test, Posterior probability = average class probability for the most likely latent class membership.

The 5-class model (in bold) was determined to have the best fit.

Supplementary Table 2: association between developmental trajectories of PE and four care pathways

|  | Developmental trajectories of self-reported psychotic experiences | | | | | | | | |
| --- | --- | --- | --- | --- | --- | --- | --- | --- | --- |
|  | Low stable (c4) | Medium stable (c5) | | Medium increasing (c1) | | Medium decreasing (c2) | | High decreasing (c3) | |
|  | (N=515, 72.4%) | (N=83; 11.7%) | | (N=37; 5.2%) | | (N=46; 6.5%) | | (N=30; 4.2%) | |
|  |  | OR | 95% CI | OR | 95% CI | OR | 95% CI | OR | 95% CI |
| 4 care pathways |  |  |  |  |  |  |  |  |  |
| End of care *(ref)* |  |  |  |  |  |  |  |  |  |
| Return to care |  | 1.27 | 0.44-3.69 | 0.63 | 0.00-4372.30 | 1.16 | 0.26-5.24 | 1.86 | 0.13-27.02 |
| Transition to AMHS |  | 1.86 | 0.85-4.08 | 2.19 | 0.78-6.17 | 0.39 | 0.04-4.38 | 3.57 | 0.82-15.55 |
| Remain in CAMHS |  | 1.63 | 0.78-3.41 | 0.38 | 0.01-10.16 | 0.50 | 0.12-2.10 | 3.57 | 0.90-14.11 |

Note. Growth mixture model with several auxiliary models (three-step approach). Models are corrected for gender and parental highest education level.
